# Impact of air pollution on life expectancy in Asian developing countries: Does renewable energy adoption matter?

**DOI:** 10.3389/fpubh.2026.1724402

**Published:** 2026-04-02

**Authors:** Bo Yang, Fatma Ahmed Hassan, Waqar Ahmad, Valentin Marian Antohi, Costinela Fortea, Cătălin Pleșea Condratovici

**Affiliations:** 1 College of Philosophy, Guizhou University, Guiyang, Guizhou, China; 2 Department of Economics, College of Business Administration, Princess Nourah bint Abdulrahman University, Riyadh, Saudi Arabia; 3 School of Economics, International Islamic University Islamabad, Islamabad, Pakistan; 4 Department of Business Administration, Dunarea de Jos University of Galati, Galati, Romania; 5 Department of Finance, Accounting and Economic Theory, Transilvania University of Brasov, Brasov, Romania; 6 Department of Morphological and Functional Sciences, Faculty of Medicine, Dunarea de Jos University of Galati, Galati, Romania

**Keywords:** air pollution, CO_2_ emissions, life expectancy, N_2_O emissions, public health, renewable energy

## Abstract

Air pollution (AIRP) remains a critical public health and environmental challenge in developing Asia, where rapid industrialization and fossil fuel dependence continue to undermine population health and longevity. This study examines the impact of AIRP on life expectancy (LIFE) and investigates whether renewable energy adoption moderates this relationship. AIRP is proxied by carbon dioxide (CO₂) and nitrous oxide (N₂O) emissions, which capture pollution-intensive energy systems, industrial activity, and agricultural practices that jointly generate health-damaging co-pollutants, particularly in contexts where consistent long-term data on ambient pollutants such as PM₂.₅ are unavailable. Using balanced panel data for 30 developing Asian countries from 2000 to 2023, we apply fixed effects, system GMM, and Lewbel IV-2SLS estimators to address unobserved heterogeneity, endogeneity, and persistence in health outcomes, while accounting for key socioeconomic and healthcare-related confounders. The results indicate that higher CO₂ and N₂O emissions significantly reduce LIFE, with CO₂ exerting a relatively stronger adverse effect. Importantly, greater renewable energy adoption weakens the negative association between emissions and LIFE, suggesting a mitigating role of clean energy transitions. While acknowledging that LIFE is influenced by multiple structural factors beyond AIRP, the findings provide robust evidence that renewable energy expansion can contribute to improved environmental quality and long-term health outcomes. These results highlight the potential co-benefits of clean energy policies for environmental sustainability and public health, offering cautious and policy-relevant insights aligned with SDG 3, SDG 7, and SDG 13.

## 1 Introduction

Air pollution (AIRP), often described as a “silent killer,” has become one of the most critical environmental and public health challenges of the 21st century. From a public health perspective, the adverse effects of AIRP operate primarily through sustained population-level exposure to polluted ambient air (1). In developing Asian economies, rapid urbanization, industrial expansion, and reliance on fossil fuels increase the concentration of harmful airborne substances, leading to prolonged inhalation exposure among large segments of the population (2, 3). According to a World Bank (4) report, nine out of ten people breathe air containing unsafe levels of pollutants in developing Asia and a notable cause of ear, nose, and throat irritations, asthma, and tachycardia in the short term, and long-term effects such as hypertension, myocardial infarction, ischemic heart disease, respiratory disorders, preterm birth, and reduced life expectancy (LIFE) (5). In 2021 alone, AIRP accounted for over 8.1 million deaths worldwide, including more than 70,000 children under five years old (6). Unlike short-term environmental shocks, AIRP constitutes a persistent health stressor, particularly in settings with limited healthcare capacity and weak environmental regulation. Consequently, AIRP represents not only an ecological externality but also a direct determinant of population health outcomes and human capital formation in developing Asia.

Geographically, the Asian continent contains almost half of the global population and faces ongoing ecological vulnerabilities. In East and the Pacific Asia, a total of 500 million children are vulnerable to AIRP, with fatalities reaching 100 children per day under 5 years of age (6). South Asia alone records severe health vulnerabilities encompassing 82,000 under-5 and 2 million premature fatalities that are tied to AIRP each year. Likewise, in central Asia, countries like Uzbekistan and Turkmenistan have health hazards tied to AIRP that are 1.5 to 2 times more severe than the global average. Around 80% of Turkmenistan’s population is forcibly breathing polluted air that exceeds the lowest interim target (7).

Greenhouse gas (GHG) emissions are a major contributor to global climate change, manifesting as extreme weather events, including global warming, prolonged droughts, rising sea levels, and other ecological disturbances (8–10). In empirical cross-country studies, AIRP is often operationalized using indicators that capture the intensity of emission-generating economic and energy systems. In this study, AIRP is proxied by carbon dioxide (CO₂) and nitrous oxide (N₂O) emissions. While these gases are widely recognized as major drivers of climate change, they also serve as comprehensive proxies for pollution-intensive production structures, fossil fuel dependence, and industrial activity that jointly contribute to ambient air quality deterioration (9, 11). Higher CO₂ and N₂O emissions reflect increased fossil fuel combustion, industrial processing, and agricultural practices, which are closely associated with elevated concentrations of harmful co-pollutants such as particulate matter (PM₂.₅), nitrogen oxides, and ozone that directly affect human health. Due to the lack of consistent, long-term, and comparable PM₂.₅ or ozone exposure data across developing Asian economies, CO₂ and N₂O emissions are employed as second-best but widely accepted proxies in the environmental health literature (12–14). This approach enables robust cross-country analysis while acknowledging that these indicators capture indirect rather than direct exposure to ambient pollutants.

**Figure 1:**
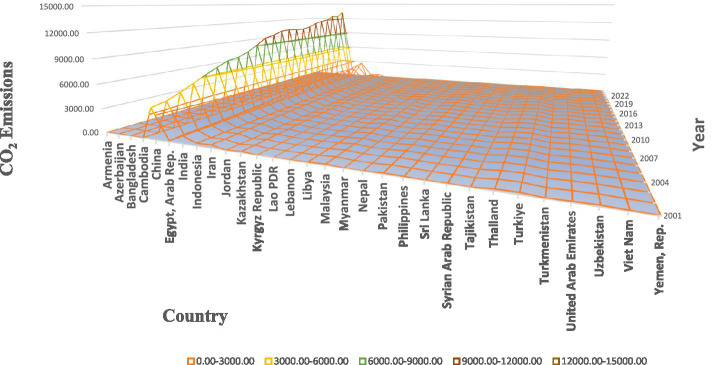
CO_2_ emissions in the selected 30 developing Asian countries. Source: author’s construction.

**Figure 2:**
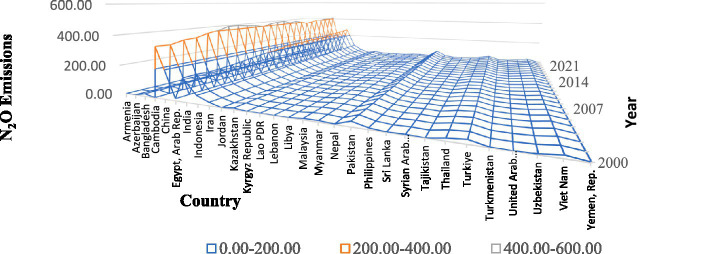
N_2_O emissions in the selected 30 developing Asian countries. Source: author’s construction.

**Figure 3:**
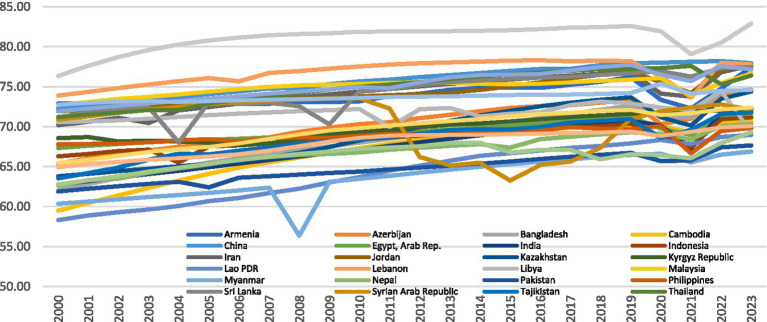
Life expectancy in the selected 30 developing Asian countries. Source: author’s construction.

Among these emissions, CO_2_ and N_2_O emissions are predominantly produced through non-renewable energy sources, contributing to environmental stress. As shown in Figure 1, CO₂ emissions in 30 developing Asian economies have exhibited a sharp upward trend from 2000 to 2023, largely driven by fossil fuel combustion and industrial growth. Similarly, Figure 2 shows the consistent rise in N₂O emissions, another major GHG generated through agricultural practices and industrial processes. Together, these pollutants deteriorate air quality and intensify climate-related risks. The result of this worsening air quality is evident in Figure 3, which demonstrates stagnating or declining LIFE trends in several Asian countries during the same period. These figures collectively portray how the pursuit of economic expansion in developing Asia has come at the expense of human health and environmental stability. Empirical evidence suggests that AIRP has already reduced average LIFE in Asia by about 2.5 to 3.3 years, greater than the global average, representing both a human and developmental cost (15, 16).

Within this context, renewable energy adoption has emerged as a vital solution for simultaneously improving environmental quality and safeguarding public health. Renewable sources such as solar, wind, hydro, and biomass generate minimal GHG emissions, thus offering a sustainable alternative to fossil-based energy systems (17). Increasing renewable energy use can substantially reduce ambient concentrations of harmful pollutants and improve population health outcomes. For instance, studies such as Alharthi et al. (18) and Aziz et al. (19) validate that expanding renewable energy capacity mitigates AIRP and decreases the disease burden associated with respiratory and cardiovascular illnesses. Hence, transitioning toward clean and sustainable energy sources not only contributes to environmental protection but also directly supports better health outcomes, thereby advancing global goals such as SDG 3 (Good Health and Well-being), SDG 7 (Affordable and Clean Energy), and SDG 13 (Climate Action).

Despite the growing evidence linking AIRP to health outcomes, existing empirical research on developing Asian countries remains limited in several respects. First, while numerous studies have documented the pollution–health nexus, no one has examined the moderating role of renewable energy adoption in this relationship. Second, most existing analyses focus on individual pollutants or specific countries, overlooking regional heterogeneity and dynamic interactions across time. Third, only a limited number of studies employ advanced econometric techniques capable of addressing endogeneity and persistence in health indicators. These gaps restrict the ability of policymakers to understand how renewable energy transitions can buffer populations against pollution-induced health risks. To bridge these gaps, this study investigates the impact of AIRP measured by CO₂ and N₂O emissions on LIFE, across 30 developing Asian countries during the period 2000–2023. It further explores whether renewable energy adoption mitigates the adverse health effects of AIRP. The main objectives are threefold: (1) to examine the direct effect of CO₂ and N₂O emissions on LIFE; (2) to analyse the moderating role of renewable energy consumption in this relationship; and (3) to provide actionable policy insights for improving air quality and health outcomes. The significance of this study lies in its integrated approach, linking environmental and health dimensions within a single empirical framework. By applying fixed effects, system GMM, and Lewbel IV-2SLS estimators, this study ensures robust inference while addressing endogeneity concerns. Overall, by integrating environmental sustainability with public health analysis, this study demonstrates that clean energy transition is not only an ecological necessity but also a crucial public health strategy. The findings aim to guide policymakers in promoting renewable energy adoption, reducing pollution exposure, and improving human lives across the developing Asian region.

## 2 Literature review

### 2.1 Air pollution and life expectancy

Previous literature has explored the impact of AIRP on human health across diverse economic landscapes. Rasoulinezhad et al. (20) and Sekhavati and Yengejeh (21) stated that fossil fuels are the main economic drivers in many countries, which has a negative influence on human health. Cakmak et al. (22) revealed that sulphur dioxide, N_2_O, carbon monoxide, and particulate matter (PM_10_ and PM_2.5_) have significantly impacted men and women in the Chilean urban centers. Wen and Gu (23) reported the same in the case of China, that AIRP harms the life span and health expectancy of Chinese elders. Specifically, women were more vulnerable than men. Similarly, Wang et al. (24) observed the harmful impact of SO_2_ and PM_10_ in southern and low-income urban areas of China. Fotourehchi (13) employed a recursive simultaneous equations model on data from 60 developing countries and found a negative impact of AIRP (PM_10_ and CO₂) on LIFE. The empirical findings of Erdoğan et al. (25) demonstrated that a unit increase in CO_2_ emissions caused an increment of 0.70% in the infant mortality rates and a decrement of 0.73 years in LIFE. Furthermore, the OLS estimations demonstrated that a unit increase in CO_2_ emissions has increased infant mortality by 0.19%, following a loss of 0.14 years in LIFE.

Likewise, Nkalu and Edeme (26) found that the consumption of solid fuel, which causes CO_2_ emissions, has significantly curtailed LIFE in Nigeria. Additionally, Lelieveld et al. (16) findings revealed that deaths attributed to AIRP are 8.8 million per year with a loss of LIFE of 2.9 years, which is double previous estimates and surpasses mortality associated with tobacco smoking. However, the global average death rate of 120 per 100,000 individuals per year has been significantly surpassed in Europe (133 per 100,000 per year) and even worse in East Asia (196 per 100,000 per year). According to Rahman et al. (14), CO_2_ emissions mainly result from production activities and have been the major cause of deteriorating ecological quality in the 31 most polluted countries of the world. Their analysis also revealed unidirectional causality from CO_2_ emission to LIFE, bidirectional causal associations between drinking water access and LIFE, as well as between sanitation services and LIFE. In addition, Adeleye et al. (27) conducted research to analyze the nexus between renewable energy-infant mortality, and CO_2_ emissions-infant mortality. Their findings revealed that renewable energy has boosted the infant mortality rate in high-income (0.11%), middle-income (0.72%), and low-income countries (0.59%). The interaction impact between real per capita income and renewable energy significantly lessens the deleterious impact of renewable energy on infant mortality under 5 years of age. Azimi and Rahman (28) investigated the impact of PM_2.5_ on health outcomes (infant mortality and LIFE) in 20 polluted nations. Their findings disclosed that the increasing PM_2.5_ levels in the air have significantly augmented infant mortality rates by 0.294% following a decline in LIFE by 3.69 years.

The empirical findings of Adebayo et al. (29) showed a negative association between CO_2_ emissions and LIFE in the United States. This impact could be mitigated by expanding health expenditures, income levels, food production, and educational attainment. In the case of Asian countries, Uddin et al. (30) found that CO_2_ emissions have significantly reduced LIFE. However, Kanat et al. (31) and Erum et al. (32) also documented the inverse relationship between AIRP and LIFE in the Republic of Kazakhstan and some polluted nations. To investigate the interplay between LIFE and CO_2_ emissions in industrialized countries, Wiredu et al. (33) performed the CS-ARDL method on data (1990–2020) and displayed a negative association between LIFE and CO_2_ emissions. Their findings recommended promoting green finance for renewable energy adoption and implementing stringent environmental regulations to improve LIFE. Furthermore, Osei-Kusi et al. (34) analyzed the energy consumption-LIFE and CO_2_ emissions-LIFE nexus in SSA, ECA, and MENA countries during the period of 1990 to 2020. Their findings revealed a negative energy consumption- LIFE and CO_2_ emissions-LIFE nexus in all countries. Mahalik et al. (35) analyzed the impact of CO_2_ emissions on LIFE across low- and middle-income countries of the world. Their findings displayed a positive association between the two in the case of low-income counties. This positive association was linked to consumption-based CO_2_ emissions, reflecting the tendency of these nations to outsource production-related emissions. Azam and Adeleye (12) reported diverse impacts of AIRP across 36 Asian and Pacific countries. Their empirical findings revealed that AIRP harms population health. In the case of SAARC economies, Guo et al. (36) reported that CO_2_ emissions adversely affected LIFE. Further, it suggested expanding health expenditures and revising their energy policies to mitigate this harmful impact.

*H1: Higher levels of air pollution are associated with increased health risks and reduced life expectancy.*

### 2.2 Renewable energy and environmental impacts

Numerous studies have explored the impact of energy consumption on AIRP in the case of different economic landscapes. Bulut (37) investigated the impact of both renewable and nonrenewable energy consumption on CO_2_ emissions. The findings revealed that renewable and nonrenewable energy have been positively associated with CO_2_ emissions. Zaidi et al. (38) in the case of Pakistan and Hasnisah et al. (39) in the case of 13 developing Asian countries, examined the association between renewable and nonrenewable energy use on CO_2_ emissions. They found a significant negative association between nonrenewable energy and CO_2_ emissions. Moreover, Hanif et al. (40) and Hanif et al. (41) reported that increasing economic growth and fossil fuel use are positively associated with CO_2_ emissions in Asian economies. Their findings recommended that renewable energy use is vitally important in mitigating AIRP. In the case of Saudi Arabia, Aziz et al. (19) found a long-term negative impact of the tech industry and renewable energy on the ecological footprint; however, the short-run impact was insignificant.

Similarly, Aziz et al. (42) examined the impact of renewable energy and globalization on the growth and environment of GCC countries. They reported a positive impact of FDI, trade, and globalization on economic growth, while renewable energy can be seen as a suitable tool to mitigate the ecological footprint associated with economic growth in the sample economies. In contrast, Yi et al. (43) investigated the effect of renewable and nonrenewable energy use on the United States’ AIRP. They revealed that both renewable and nonrenewable energy sources have been positively associated with AIRP because renewable energy has not reached its minimum level to mitigate CO_2_ emissions. Another empirical study by Li et al. (44) evaluated the impact of renewable energy consumption on AIRP in China. Results revealed that renewable energy significantly reduced economic growth boosted ecological footprint. Alharthi et al. (18) studied the nexus between renewable energy use and AIRP in MENA countries. Their findings showed that the increasing use of renewable energy is pivotal in reducing reliance on nonrenewable energy sources and providing better air quality.

*H2: Higher levels of renewable energy use weaken the adverse impact of air pollution on public health and increase life expectancy.*

### 2.3 Gaps in existing literature and contribution of this study

Despite extensive research on AIRP, renewable energy, and health outcomes, three key gaps persist in the literature. First, few studies explicitly distinguish between pollution as an environmental outcome and pollution as a health exposure mechanism, particularly in macro-level empirical models. Second, while LIFE is increasingly recognized as a robust indicator of cumulative health impacts, it remains underutilized in studies examining energy transitions and pollution exposure. Third, the moderating role of renewable energy in the pollution–health relationship has largely been overlooked, with existing research focusing either on direct pollution effects or on environmental sustainability outcomes in isolation.

This study addresses these gaps by integrating AIRP, renewable energy adoption, and LIFE within a unified empirical framework. By examining whether renewable energy consumption mitigates the adverse impact of AIRP on LIFE across 30 developing Asian countries, this research advances the environmental health literature beyond descriptive associations. The use of dynamic panel estimators further strengthens causal inference, offering novel evidence on renewable energy as both an environmental and public health policy instrument.

## 3 Theoretical framework

The theoretical foundation of this study is grounded in the Health Production Function (HPF) developed by Grossman (45), which provides a microeconomic framework for analyzing health as an outcome of various socio-economic and environmental inputs. It conceptualizes health as both a consumption good, generating immediate utility, and an investment good, enhancing productivity and future well-being. According to HPF, health outcomes are influenced by positive inputs such as income, education, healthcare access, and environmental quality, while detrimental factors, particularly pollution, degrade health capital. Thus, individuals and societies allocate resources to produce and maintain health, whereas adverse determinants reduce its stock and compromise outcomes (12, 46, 47). Formally, the HPF can be expressed as:


$$\text{Health Outcomes}=f\;(\begin{array}{l} \mathrm{Air}\;\text{Pollution},\text{Renewable Energy},\\ \text{Control Variables} \end{array})$$

**Figure 4:**
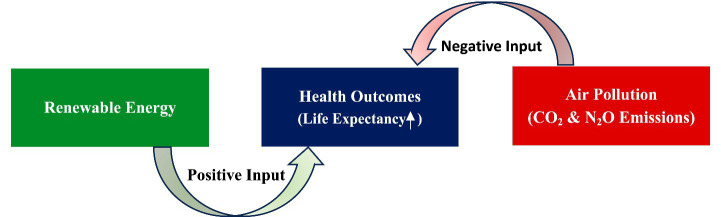
Health production function. Source: author’s construction.

In this study, AIRP is proxied by CO₂ and N₂O emissions, representing harmful environmental inputs that deteriorate health capital. Higher levels of these pollutants contribute to respiratory and cardiovascular diseases, thereby reducing LIFE (48). Conversely, renewable energy adoption serves as a protective input in the production process, enhancing environmental quality and mitigating pollution-induced health risks without constraining economic growth. This relationship is especially relevant for developing Asian economies, where rapid industrialization and heavy dependence on fossil fuels intensify exposure to AIRP. In such contexts, limited institutional and healthcare capacities further heighten vulnerability to pollution-related health challenges. Figure 4 also presents the HPF.

**Figure 5:**
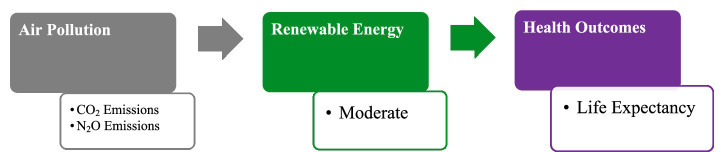
Role of renewable energy in improving health outcomes. Source: author’s construction.

The HPF explains how several inputs influence the loss of health capital. Based on these perspectives, the pollution-health nexus particularly highlights a direct route whereby CO_2_ and NO_2_ emissions significantly bring about destructive health outcomes. In this paradigm, renewable energy appears to be a critical factor, which could reduce CO_2_ and N_2_O emissions, ultimately contributing to public health outcomes as presented in Figure 5. This moderating mechanism implies that the negative impact of AIRP on LIFE is conditional on a country’s energy structure. In economies with higher renewable energy shares, pollution-related health risks are expected to be lower due to reduced exposure intensity and improved air quality. Conversely, in fossil fuel–dominated energy systems, pollution exposure remains high, amplifying its detrimental effects on population health. Accordingly, this study hypothesizes that renewable energy consumption attenuates the adverse effect of AIRP on LIFE in developing Asian economies.

This framework also accommodates the control variables, such as income (41), population growth (30), fossil fuel consumption (40), and health expenditures (12), which reflect both the efficacy of health systems and the extent to which populations are exposed to environmental deterioration. For instance, high income levels and health expenditures expand access to medical services and nutrition. In contrast, population growth and fossil fuel consumption intensify exposure to pollution and amplify the deleterious impacts of environmental degradation. This framework unfolds the important role of renewable energy adoption in moderating the impact of AIRP on LIFE in the developing Asian economies.

Although CO₂ and N₂O emissions are not classified as direct toxic air pollutants like PM2.5 or NO₂, their association with LIFE operates through well-established indirect climate–health pathways. According to the World Health Organization (49), climate change represents one of the most significant global health threats, contributing to increased heat-related mortality, cardiovascular and respiratory stress, dehydration, kidney disease, and the expansion of vector-borne diseases such as malaria and dengue. Romanello et al. (50) further document rising heatwave exposure among older adults, increased malnutrition risks, and growing health burdens linked to extreme weather events. In this context, GHG emissions affect population health through systemic environmental changes rather than acute toxicity. Moreover, CO₂ emissions are closely linked to fossil fuel combustion, which simultaneously releases harmful co-pollutants such as particulate matter, sulfur dioxide, and nitrogen oxides, pollutants directly associated with premature mortality and chronic diseases (World Health Organization, 2021). Thus, CO₂ serves as a macro-level indicator of carbon-intensive development and broader environmental degradation. Similarly, N₂O emissions, largely originating from agricultural and industrial activities, contribute to climate change and ozone depletion, indirectly increasing health risks through ecosystem disruption, food insecurity, and ultraviolet radiation exposure. Therefore, the negative association between greenhouse gas emissions and LIFE should be interpreted within a climate-mediated and pollution-linked health framework, consistent with contemporary epidemiological evidence.

In conclusion, the theoretical framework thus establishes a conditional relationship in which AIRP adversely affects LIFE, while renewable energy moderates this effect by reducing exposure to health-damaging emissions. This conceptualization directly informs the empirical model, where LIFE is specified as a function of AIRP, renewable energy consumption, their interaction term, and a set of socioeconomic control variables. The inclusion of the interaction term captures the hypothesized exposure-reduction mechanism and allows for empirical testing of renewable energy as both an environmental and public health policy instrument.

## 4 Data and methodology

### 4.1 Data and variables

This study employs a balanced panel dataset covering 30 developing Asian countries over the period 2000–2023 to empirically test the proposed hypotheses. The choice of countries and the study period is determined by data availability. The analysis begins in 2000, the earliest year for which continuous and comparable data exist, and extends to 2023, constrained by the availability of recent data, particularly on AIRP indicators. Developing Asia is selected as the focus of this study because it provides a relevant context for examining the stated objectives. These economies are characterized by severe AIRP, a high prevalence of health-related challenges, and reliance on low-technology energy sources, making them an ideal setting for such an investigation.

LIFE is the dependent variable, which represents the average number of years a newborn infant is projected to live, assuming the age-specific mortality rate to be constant throughout its lifespan. Numerous studies have used this indicator in demographic, economic, and environmental studies to assess public health (16, 23, 24).

AIRP is an explanatory variable in the models proxied by CO_2_ and N_2_O emissions due to their consistent availability and comparability across developing Asian economies over the study period. While these indicators are primarily classified as GHG rather than direct ambient pollutants, they serve as comprehensive proxies for fossil fuel–intensive energy systems, industrial activity, and agricultural practices that jointly generate health-damaging co-pollutants such as particulate matter (PM₂.₅), nitrogen oxides, and sulfur dioxide. From a public health exposure perspective, higher CO₂ and N₂O emissions reflect production and energy structures associated with elevated population exposure to polluted air. The direct inclusion of PM₂.₅ or ozone exposure indicators was not feasible due to substantial data gaps, inconsistent measurement methodologies, and limited temporal coverage across the sampled countries. Consequently, CO₂ and N₂O emissions are employed as second-best proxies, a practice widely adopted in cross-country environmental health studies where direct exposure data are unavailable. This limitation is explicitly acknowledged when interpreting the results, which should be understood as reflecting pollution-intensive economic activity rather than precise ambient exposure levels. These gases mainly result from different industrial and manufacturing processes. For instance, CO_2_ emissions mainly result from different industrial and manufacturing processes, such as cement manufacturing processes, utilization of solvents, and other chemical processes, often energy-intensive processes that release substantial amounts of CO_2_ emissions into the air (2, 9). Likewise, N_2_O emissions mainly result from chemical manufacturing processes such as adipic and nitric acid production and agricultural fertilizer use, and soil management (51).

Renewable energy consumption (REC) acts as a moderating variable in this study. It is considered an important factor to mitigate AIRP because it is derived from renewable sources such as solar, wind, geothermal, etc., without depleting natural resources. It represents a country’s transition toward a clean energy system. However, the increasing share of REC in the total energy mix contributes to a substantial decline in pollution emissions and promotes sustainable development (9). Inspired by existing related literature, our models include the health expenditure (HEXP), fossil fuel energy consumption (FFEC), population density (POPD), GDP per capita (GDPPC), and hospital beds (HBED) as control variables (29, 31). Table 1 presents the variables’ description and source.

**Table 1:** Variable description and source.

Variables	Descriptions	Expected signs	Source
Dependent variable			
Life Expectancy (LIFE)	Log of life expectancy at birth, total (years)		WDI, WB
Explanatory variable			
Carbon Dioxide Emissions (CO_2_)	Log of CO_2_ emissions, total (Mt CO2e)	−	WDI, WB
Nitrous Oxide Emissions (N_2_O)	Log N_2_O emissions, total (Mt CO2e)	−	WDI, WB
Moderating variable			
Renewable Energy (REC)	Renewable energy consumption (% of total final energy consumption)	+	WDI, WB
Control variable			
Health Expenditure (HEXP)	Current health expenditure (% of GDP)	+	WDI, WB
Fossil Fuel Energy Consumption (FFEC)	Fossil fuel energy consumption (% of total)	−	WDI, WB
Population Density (POPD)	Log of population density (people per sq. km of land area)	−	WDI, WB
GDP *Per Capita* (GDPPC)	GDP per capita (annual % growth)	+	WDI, WB
Hospital Beds (HBED)	Hospital beds (per 1,000 people)	+	WDI, WB

WDI, World Development Indicators; WB, World Bank.

### 4.2 Model specifications

Our empirical strategy proceeds in two steps. First, we estimate the direct effect of the AIRP on LIFE. Second, we assess the moderating role of REC in the nexus between AIRP and LIFE. The baseline dynamic panel model is specified as:


$$\mathrm{LIF}E_{\mathrm{it}}=\alpha_{0}+\alpha_{1}\mathrm{LIF}E_{\mathrm{it}-1}+\alpha_{2}\mathrm{AIR}P_{\mathrm{it}}+\alpha \prime Z_{\mathrm{it}}+w_{i}+v_{t}+\mu_{\mathrm{it}}\;\;$$ (1)

Where LIFE denotes the life expectancy. AIRP represents the air pollution measured by CO_2_ and N_2_O emissions. Z is a vector of control variables. $w_{i}$ and $v_{t}$ capture country and time-fixed effects, respectively; and $\mu_{\mathrm{it}}$ is the error term. Equation 1 is operationalized separately for CO_2_ and N_2_O emissions:


$$\mathrm{LIF}E_{\mathrm{it}}=\alpha_{0}+\alpha_{1}\mathrm{LIF}E_{\mathrm{it}-1}+\alpha_{2}\mathrm{CO}2_{\mathrm{it}}+\alpha \prime Z_{\mathrm{it}}+w_{i}+v_{t}+\mu_{\mathrm{it}}\;$$ (2)

$$\mathrm{LIF}E_{\mathrm{it}}=\alpha_{0}+\alpha_{1}\mathrm{LIF}E_{\mathrm{it}-1}+\alpha_{2}N2O_{\mathrm{it}}+Z+w_{i}+v_{t}+\mu_{\mathrm{it}}\;\;$$ (3)

To examine the moderating role of REC in the nexus between CO_2_ emissions and LIFE, we include interaction terms between CO_2_ and REC. Equation 2 is extended as:


$$\begin{array}{l} \mathrm{LIF}E_{\mathrm{it}}=\beta_{0}+\beta_{1}\mathrm{LIF}E_{\mathrm{it}-1}+\beta_{2}\mathrm{CO}2_{\mathrm{it}}+\beta_{3}(\mathrm{CO}2_{\mathrm{it}}\times \mathrm{RE}C_{\mathrm{it}})\\ +\alpha \prime Z_{\mathrm{it}}+w_{i}+v_{t}+\mu_{\mathrm{it}} \end{array}$$ (4)

Similarly, to explore the moderating role of REC in the nexus between N_2_O emissions and LIFE, we include interaction terms between N_2_O and REC. Equation 3 is extended as:


$$\begin{array}{l} \mathrm{LIF}E_{\mathrm{it}}=\beta_{0}+\beta_{1}\mathrm{LIF}E_{\mathrm{it}-1}+\beta_{2}N2O_{\mathrm{it}}+\beta_{3}(N2O_{\mathrm{it}}\times \mathrm{RE}C_{\mathrm{it}})\\ +\alpha \prime Z_{\mathrm{it}}+w_{i}+v_{t}+\mu_{\mathrm{it}} \end{array}$$ (5)

To explore the marginal effects of CO_2_ emissions on LIFE, under various levels of REC, we take the partial derivative of Equation 4 with respect to CO_2_ emissions.


$$\frac{\partial (\mathrm{LIF}E_{\mathrm{it}})}{\partial (\mathrm{CO}2_{\mathrm{it}})}=\beta_{2}+\beta_{3}\mathrm{RE}C_{\mathrm{it}}$$ (6)

Equation 6 represents the marginal impact of CO2 emissions on LIFE.

Similarly, to explore the marginal effects of N_2_O emissions on LIFE, under various levels of REC, we take the partial derivatives of Equation 5 with respect to N_2_O emissions.


$$\frac{\partial (\mathrm{LIF}E_{\mathrm{it}})}{\partial (N2O_{\mathrm{it}})}=\beta_{2}+\beta_{3}\mathrm{RE}C_{\mathrm{it}}$$ (7)

Equation 7 represents the marginal impact of N_2_O emissions on LIFE.

### 4.3 Estimation methods

This study employs the fixed effects (FE), system GMM and Lewbel IV-2SLS estimators to empirically assess the relationship between AIRP and LIFE, and to explore the moderating role of renewable energy. Using a combination of estimation techniques enhances the robustness of inference and strengthens the empirical credibility of the results. Initially, both FE and random effects (RE) models are estimated. To select the appropriate model, the Hausman (52) test is conducted, which compares the efficiency and consistency of the two estimators. The test rejects the null hypothesis that the regressors are uncorrelated with country-specific effects, confirming the suitability of the FE estimator. The FE estimator effectively controls for time-invariant unobserved heterogeneity across countries, such as geographic, institutional, and structural differences that could otherwise bias the estimates (53). Country and time FE are incorporated to capture unobserved characteristics and global shocks common to all countries in a given year, including pandemics, international environmental agreements, and global economic crises.

However, the FE model relies on the assumption of strict exogeneity, which may be violated when key explanatory variables such as pollution indicators and REC are endogenous. For instance, deteriorating health outcomes may encourage policy actions toward cleaner energy use or increase public healthcare spending, creating reverse causality. Additionally, omitted variable bias and measurement errors, especially in environmental and health data, may compromise the validity of FE estimates (54). To overcome these econometric challenges, we apply the system GMM estimator, developed by Arellano and Bover (55) and Blundell and Bond (56). This estimator is particularly suitable for dynamic panel data characterized by a relatively large cross-sectional dimension and a moderate time dimension, as in our case of 30 developing Asian economies observed from 2000 to 2023. System GMM addresses issues of endogeneity, simultaneity bias, feedback effects, and unobserved heterogeneity by estimating a system of equations in both first differences and levels, using lagged levels of the variables as instruments for the differenced equations and lagged differences as instruments for the level equations (57).

The inclusion of the lagged dependent variable, LIFE captures the dynamic nature of health outcomes, which evolve gradually over time due to persistence in health infrastructure and environmental exposure. The system GMM method is implemented using the “xtabond2” command in Stata, with the robust option to produce heteroskedasticity and autocorrelation consistent standard errors. This approach ensures the accuracy of inference even in the presence of mild violations of classical assumptions. To verify the robustness and internal validity of the GMM estimates, several diagnostic tests are performed. The Arellano–Bond AR (1) and AR(2) tests check for the absence of second-order serial correlation in the residuals, while the Hansen and Sargan tests assess the validity of the instruments. The F-statistics further confirm the joint significance of the regressors, ensuring that the explanatory variables collectively explain variations in health outcomes.

To further address potential endogeneity concerns, particularly reverse causality, we implement an additional robustness check using the heteroskedasticity-based instrumental variable approach proposed by Lewbel (58). While the system GMM estimator mitigates endogeneity by employing internal instruments derived from lagged variables, it relies on moment conditions that may not fully eliminate bias arising from unobserved time-varying confounders. The Lewbel IV approach generates internal instruments based on model heteroskedasticity, exploiting higher-moment conditions to construct instruments without requiring external exclusion restrictions. This method is particularly suitable in cross-country panel settings where credible external instruments are difficult to identify. Following Lewbel (58), we generate instruments using mean-centered exogenous variables interacted with model residuals, ensuring identification under the assumption that the error term exhibits heteroskedasticity.

**Figure 6:**
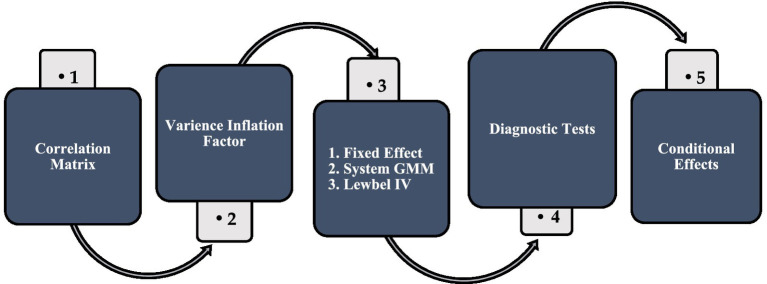
Estimation flowchart. Source: author’s construction.

The consistency of results across FE, system GMM, and Lewbel IV estimations strengthens confidence in the causal interpretation of the findings. The estimation flowchart is also present in Figure 6.

## 5 Results

### 5.1 Descriptive statistics

Table 2 presents the descriptive statistics for all the variables in the analysis. The average LIFE is 70.72 years, with a minimum and maximum ranging between 56.36 and 82.91 years, suggesting fair variation across countries. CO₂ emissions and N₂O emissions have average values along with standard deviations of 477.27 (SD = 1716.71) and 31.72 (SD = 79.13), respectively, suggesting substantial variability in industrialization, fossil fuel combustion, and economic development. REC averages 24.65% (SD = 25.74), with minimum and maximum values ranging from 0 to 91.30, suggesting a relatively small share of renewable energy in total energy use. The average HEXP is 24.65% of GDP (SD = 1.81), with minimum and maximum values ranging between 1.77 and 12.32%, indicating distinct disparities in prioritizing health expenditures. Moreover, the average values of FFEC, POPD, GDPPC, and HBED are 76.83% (SD = 24.38), 173.55 (SD = 226.12), 3.36 (SD = 6.68), and 2.45 (SD = 1.78), respectively, demonstrating highly urbanized, densely populated, heavy dependence on fossil fuels, and diverse income and health care infrastructure across selected Asian countries.

**Table 2:** Summary statistics.

Variables	LIFE	CO_2_	N_2_O	REC	HEXP	FFEC	POPD	GDPPC	HBED
Obs.	720	720	720	720	720	720	720	720	720
Mean	70.72	477.27	31.72	24.65	4.59	76.83	173.55	3.36	2.45
Standard Dev.	4.63	1716.71	79.13	25.74	1.81	24.38	226.12	6.68	1.78
Minimum	56.36	0.93	0.29	0.00	1.77	8.61	3.02	−49.13	0.10
Maximum	82.91	13259.64	427.02	91.30	12.32	100	1301.26	91.78	7.62

### 5.2 Correlation estimates

The correlation results in Table 3 indicate that CO₂ emissions and N₂O emissions are both strongly and negatively associated with LIFE. This suggests that higher levels of CO_2_ and N_2_O emissions significantly reduce LIFE in Asian developing economies, reflecting the detrimental health effects of AIRP. Conversely, REC shows a significant positive correlation with LIFE, implying that greater reliance on clean and renewable energy sources is linked to improved public health outcomes. Furthermore, renewable energy is negatively correlated with both CO₂ and N₂O emissions, indicating that the adoption of renewable energy contributes to lowering harmful emissions. Overall, these relationships highlight that promoting REC not only mitigates AIRP but also enhances population health and longevity.

**Table 3:** Correlation matrix.

Variables	LIFE	CO_2_	N_2_O	REC	HEXP	FFEC	GDPPC	POPD	HBED
LIFE	1								
CO2	−0.51***	1							
	(0.003)								
N2O	−0.42***	0.41***	1						
	(0.005)	(0.000)							
REC	0.31**	−0.48***	−0.41***	1					
	(0.021)	(0.012)	(0.000)						
HEXP	0.69***	0.61***	0.31**	−0.19**	1				
	(0.000)	(0.000)	(0.043)	(0.041)					
FFEC	−0.34**	0.71***	0.41*	−0.62**	0.51**	1			
	(0.042)	(0.000)	(0.072)	(0.052)	(0.025)				
GDPPC	0.57***	0.26**	0.35**	0.49***	0.66***	0.51***	1		
	(0.000)	(0.046)	(0.043)	(0.000)	(0.000)	(0.005)			
POPD	−0.33**	0.31**	0.18*	0.17	0.32***	0.39**	0.26*	1	
	(0.051)	(0.031)	(0.061)	(0.213)	(0.003)	(0.038)	(0.062)		
HBED	0.28**	0.14	0.23**	−0.24	0.77***	0.14	0.29***	0.56**	1
	(0.051)	(0.126)	(0.039)	(0.137)	(0.000)	(0.191)	(0.000)	(0.044)	

*p*-values in parentheses. *, **, *** show 10, 5, 1% level of significance, respectively.

### 5.3 Multicollinearity test

Table 4 presents the results of the multicollinearity test using the Variance Inflation Factor (VIF). The VIF values range from 1.87 to 4.13, all well below the conventional threshold of 10, indicating the absence of serious multicollinearity among the explanatory variables. This suggests that the independent variables are not highly correlated with one another, and thus, the estimated regression coefficients are likely to be statistically reliable and robust. Consequently, the regression results can be interpreted with confidence, free from concerns of multicollinearity.

**Table 4:** Variance inflation factors.

Variables	VIF	1/VIF
CO_2_	3.78	0.29
N_2_O	4.13	0.24
REC	3.13	0.32
HEXP	2.32	0.43
FFEC	1.87	0.53
GDPPC	2.38	0.42
POPD	1.98	0.51
HBED	2.85	0.35

### 5.4 Regression analysis

#### 5.4.1 Impact of CO_2_ and N_2_O emissions on life expectancy

The results in Table 5 reveal the impact of both CO_2_ and N_2_O emissions on LIFE employing both FE and system GMM. The estimates are consistent across both specifications; however, there are notable variations regarding controlling endogeneity. The lagged dependent coefficient of LIFE (−1) is highly significant and positive (0.945 for CO_2_ and 0.895 for N_2_O models) under the system GMM specification. This underscores persistence in LIFE and emphasizes the use of the system GMM to capture dynamic feedback effects and path dependency. Most importantly, both AIRP indicators have a negative impact on LIFE under all estimators. In FE models, CO_2_ and N_2_O emissions (− 0.245, − 0.121) indicate a significantly negative impact on LIFE. In addition, this impact intensifies under the system GMM model (CO_2_ = −0.311 and N_2_O = −0.198), reflecting the fact that, after addressing dynamic bias and endogeneity, the impact of AIRP on LIFE becomes stronger. For instance, a unit increase in CO_2_ emissions reduces LIFE by 0.311 years (≈3.7 months). Likewise, a corresponding increase in N_2_O emissions lowers LIFE by 0.198 years (≈2.4 months).

**Table 5:** Impact of CO_2_ and N_2_O emissions on life expectancy.

Dep. Var: LIFE	Fixed effects		System GMM	
	CO_2_	N_2_O	CO_2_	N_2_O
LIFE (−1)			0.945***	0.895***
			(0.121)	(0.142)
CO2	−0.245***		−0.311**	
	(0.094)		(0.151)	
N2O		−0.121**		−0.198***
		(0.061)		(0.042)
HEXP	0.211**	0.275***	0.178*	0.217***
	(0.087)	(0.061)	(0.095)	(0.034)
FFEC	−0.101**	−0.137	−0.165**	−0.121*
	(0.045)	(0.089)	(0.078)	(0.070)
GDPPC	0.211***	0.172**	0.110**	0.127***
	(0.065)	(0.075)	(0.045)	(0.016)
POPD	−0.088*	−0.092	−0.124*	−0.111
	(0.052)	(0.068)	(0.068)	(0.076)
HBED	0.111**	0.145***	0.089*	0.092*
	(0.049)	(0.037)	(0.048)	(0.054)
Constant	5.217***	4.854***	3.617**	2.809**
	(0.989)	(0.721)	(1.487)	(1.401)
Diagnostics checks				
Observations	720	720	690	690
Countries	30	30	30	30
R-squared	0.713	0.671		
Hausman Test	26.32**	29.21**		
Country FE	Yes	Yes	Yes	Yes
Time FE	Yes	Yes	Yes	Yes
Instruments			16	19
F-test			27.98	31.83
AR1			0.000	0.000
AR2			0.478	0.254
Hansen			0.398	0.423
Sargan			0.313	0.299

Standard errors in parentheses. *, **, *** show 10, 5, 1% level of significance, respectively.

In FE models, HEXP coefficients are significantly positive (0.211 and 0.275) and remain robust in the system GMM model (0.178 and 0.217). This suggests that increasing HEXP plays a major role in health outcomes. FFEC is significantly negative across specifications. For instance, in the FE model, the FFEC coefficients are 0.101 and −0.137, whereas in the system GMM model, the coefficients are −0.165 and −0.121. This underscores that fossil fuels not only contribute to emission levels but also produce negative health outcomes. Likewise, GDPPC reveals a positive and significant relationship with LIFE across all models. The coefficients (0.211, 0.172) in the FE models and (0.110, 0.127) in the System GMM models confirm this association. This indicates that higher income levels contribute to improved public health and longevity in Asian developing economies. POPD is negatively signed across all models, implying that demographic burdens also contribute to devastating health outcomes. Moreover, HBED shows a positive and significant relationship with LIFE in both FE (0.111, 0.145) and system GMM (0.089, 0.092) models. This indicates that improved healthcare infrastructure and greater hospital capacity contribute to better health outcomes. The results suggest that increasing hospital bed availability plays a vital role in enhancing LIFE in Asian developing economies.

The diagnostic checks confirm the robustness and validity of the estimated models. The Hausman test values (26.32 and 29.21) support the use of the FE model over RE, indicating consistent estimators. In the system GMM estimations, the AR (1) test is significant while the AR (2) is not, confirming the absence of second-order autocorrelation. Furthermore, the Hansen and Sargan test *p*-values (above 0.10) indicate that the instruments used are valid and not over-identified. Overall, these diagnostics confirm that the models are statistically sound and the results are reliable.

#### 5.4.2 Moderating role of renewable energy in the Nexus between CO_2_, N_2_O, and life expectancy

The results in Table 6 present the moderating role of renewable energy use in the relationship between AIRP and LIFE. The coefficient of the lagged dependent variable, LIFE (−1), is positive and highly significant in the system GMM models, indicating that LIFE is persistent over time; countries with higher LIFE in one period tend to maintain it in subsequent years. Moreover, both CO₂ (−0.317, −0.355) and N₂O (−0.211, −0.197) emissions indicate significant negative effects on LIFE under all model specifications, confirming that higher AIRP levels severely deteriorate population health in Asian developing economies. Crucially, the interaction terms, CO₂ × REC (−0.021, −0.012) and N₂O × REC (−0.044, −0.019) are statistically significant and negative across both FE and system GMM models. These results demonstrate that renewable energy adoption effectively mitigates the adverse impact of AIRP on LIFE. The greater use of renewable energy sources offsets part of the health damage caused by CO₂ and N₂O emissions, underscoring its protective and moderating role in improving environmental and health outcomes. Renewable energy adoption may not operate as a direct health intervention per se but rather as a proxy for broader structural transformations, including cleaner energy systems, improved environmental regulation, technological upgrading, and enhanced institutional capacity. These complementary changes jointly contribute to reduced emission intensity and lower population exposure to health-damaging pollutants.

**Table 6:** Moderating role of renewable energy in the nexus between CO_2_, N_2_O, and life expectancy.

Dep. Var: LIFE	Fixed effects		System GMM	
	CO_2_	N_2_O	CO_2_	N_2_O
LIFE (−1)			0.789***	0.911***
			(0.124)	(0.214)
CO2	−0.317***		−0.355***	
	(0.027)		(0.098)	
N2O		−0.211***		−0.197**
		(0.009)		(0.088)
CO2 × REC	−0.021***		−0.012**	
	(0.007)		(0.006)	
N2O × REC		−0.044**		−0.019**
		(0.019)		(0.009)
HEXP	0.204**	0.178**	0.221***	0.127*
	(0.089)	(0.089)	(0.078)	(0.071)
FFEC	−0.097**	−0.035	−0.131**	−0.110**
	(0.041)	(0.050)	(0.062)	(0.045)
GDPPC	0.184***	0.134**	0.189**	0.231**
	(0.049)	(0.069)	(0.078)	(0.099)
POPD	−0.087*	−0.125	0.133*	0.074*
	(0.049)	(0.089)	(0.077)	(0.041)
HBED	0.092	0.122**	0.119**	0.099*
	(0.068)	(0.051)	(0.058)	(0.052)
Constant	4.254***	7.148***	2.501**	1.876*
	(0.868)	(0.824)	(0.999)	(0.988)
Diagnostics checks				
Observations	720	720	690	690
Countries	30	30	30	30
R-squared	0.593	0.662		
Hausman Test	21.82**	26.35***		
Country FE	Yes	Yes	Yes	Yes
Time FE	Yes	Yes	Yes	Yes
Instruments			21	19
F-test			23.13	19.23
AR1			0.000	0.000
AR2			0.372	0.293
Hansen			0.398	0.281
Sargan			0.661	0.562

Standard errors in parentheses. *, **, *** show 10, 5, 1% level of significance, respectively.

Among the control variables, HEXP, GDPPC, and HBED positively contribute to LIFE, while FFEC and POPD show negative effects. The diagnostic checks confirm the reliability of the estimated models. The Hausman test justifies the use of the FE estimator. In the system GMM models, the AR (1) test is significant while the AR (2) is not, indicating no second-order serial correlation. Moreover, the Hansen and Sargan test p-values confirm that the instruments used are valid and not overidentified, ensuring the robustness of the results.

#### 5.4.3 Marginal effects of CO_2_ and N_2_O emissions on life expectancy at different levels of renewable energy consumption

The results in Table 7 present the marginal effects of CO₂ and N₂O emissions on LIFE across varying levels of REC using both FE and system GMM estimators. At low levels of REC (25th percentile), the coefficients of CO₂ (−0.197, −0.261) and N₂O (−0.151, −0.165) are negative and statistically significant, indicating that increased emissions substantially reduce LIFE. Specifically, a unit rise in CO₂ and N₂O emissions lowers LIFE by 0.261 years (≈3.1 months) and 0.165 years (≈2 months), respectively. This reflects that those countries highly dependent on fossil fuels, with minimal renewable energy integration, suffer the most severe health consequences from AIRP. At a moderate level of REC (50th percentile), the adverse effects of CO₂ (−0.087, −0.119) and N₂O (−0.093, −0.101) emissions on LIFE remain significant but noticeably weaker. This reduction in the magnitude of negative impacts implies that as renewable energy use increases, cleaner energy sources begin to offset pollution-induced health damages.

**Table 7:** Marginal effects of CO_2_ and N_2_O emissions on life expectancy at different levels of renewable energy.

Percentile levels	Fixed effects		System GMM	
	CO_2_	N_2_O	CO_2_	N_2_O
REC at P25	−0.197***	−0.151*	−0.261***	−0.165**
	(0.034)	(0.087)	(0.034)	(0.071)
REC at P50	−0.087**	−0.093**	−0.119***	−0.101**
	(0.038)	(0.045)	(0.037)	(0.045)
REC at P75	−0.011***	−0.048***	−0.039***	−0.054**
	(0.005)	(0.018)	(0.009)	(0.027)

P25, P50, and P75 are the 25th, 50th and 75th percentiles, respectively. ***p-values <0.01, **p-value <0.05, and *p-value <0.1.

**Figure 7:**
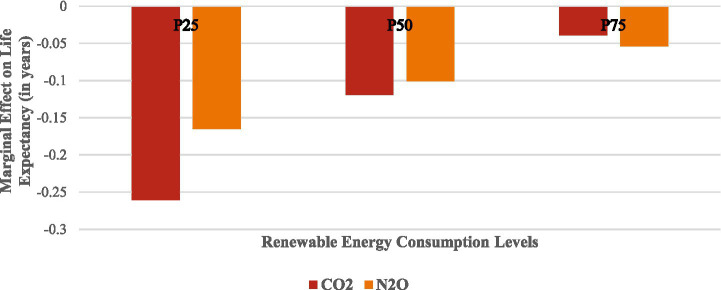
Marginal impact of CO_2_ and N_2_O emissions on LIFE at different levels of REC (system GMM results).

At the high level of REC (75th percentile), the harmful effects of CO₂ and N₂O emissions further decline, reducing LIFE by only 0.039 years (≈0.46 months) and 0.054 years (≈0.65 months), respectively. This demonstrates a strong mitigating effect of renewable energy adoption, suggesting that greater reliance on renewables substantially cushions populations from pollution-related health deterioration. Overall, these findings confirm that the negative health impacts of AIRP are inversely related to renewable energy intensity. As countries transition toward cleaner energy portfolios, the detrimental effects of CO₂ and N₂O emissions on LIFE are significantly reduced. Figure 7 also presents the marginal impact results.

#### 5.4.4 Robustness check

The results in Table 8 provide additional robustness checks using the Lewbel IV-2SLS estimator to address potential reverse causality and unobserved time-varying confounders. The Lewbel approach constructs internal instruments based on heteroskedasticity, thereby strengthening identification beyond the dynamic system GMM framework. Consistent with the baseline FE and system GMM results, CO₂ emissions continue to have a statistically significant negative effect on LIFE. Specifically, the CO₂ coefficient (−0.189, *p* < 0.05) indicates that a unit increase in emissions reduces LIFE by approximately 0.189 years. Likewise, N₂O emissions remain negatively associated with LIFE (−0.118, *p* < 0.10) in the baseline model, confirming that AIRP deteriorates LIFE outcomes even after controlling for potential endogeneity. Importantly, the interaction terms between emissions and REC remain negative and statistically significant. The coefficients for CO₂ × REC (−0.011, *p* < 0.05) and N₂O × REC (−0.019, *p* < 0.05) confirm that renewable energy adoption significantly mitigates the adverse health effects of pollution. These findings reinforce the moderating role of REC observed in the system GMM estimates and suggest that cleaner energy transitions reduce pollution-induced health damages.

**Table 8:** Lewbel IV-2SLS robustness results.

Dep. Var: LIFE	CO_2_	N_2_O	CO_2_ × REC	N_2_O × REC
CO_2_	−0.189**		−0.176***	
	(0.073)		(0.058)	
N_2_O		−0.118*		0.131***
		(0.067)		(0.039)
CO_2_ × REC			−0.011**	
			(0.006)	
N_2_O × REC				−0.019**
				(0.009)
HEXP	0.110**	0.172**	0.042**	0.211***
	(0.045)	(0.075)	(0.019)	(0.065)
FFEC	−0.165**	−0.037*	−0.136**	−0.101**
	(0.078)	(0.021)	(0.061)	(0.045)
GDPPC	0.121*	0.134**	0.137	0.097**
	(0.070)	(0.069)	(0.089)	(0.041)
POPD	−0.042**	0.092	−0.111	−0.088*
	(0.019)	(0.068)	(0.076)	(0.052)
HBED	0.089	0.184***	0.096*	0.091*
	(0.063)	(0.049)	(0.053)	(0.052)
Constant	2.501**	2.809**	3.284**	2.917**
	(0.999)	(1.401)	(1.302)	(1.188)
Diagnostics checks				
Observations	720	720	720	720
Countries	30	30	30	30
Country FE	Yes	Yes	Yes	Yes
Time FE	Yes	Yes	Yes	Yes
Kleibergen–Paap F-stat	16.98	18.25	19.25	21.27
Endogeneity Test	0.019	0.016	0.021	0.018
Hansen	0.345	0.472	0.287	0.571

Standard errors in parentheses. *, **, *** show 10, 5, 1% level of significance, respectively.

Regarding control variables, HEXP, GDPPC, and HBED generally retain positive and significant effects on LIFE, whereas FFEC and POPD show negative associations. This consistency across estimation techniques strengthens confidence in the structural validity of the model. The diagnostic statistics further support the credibility of the IV estimates. The Kleibergen–Paap F-statistics range between 16.98 and 21.27, exceeding the conventional threshold of 10, indicating strong instrument relevance. The endogeneity tests are statistically significant, justifying the use of instrumental variable techniques. Moreover, the Hansen test *p*-values (0.287–0.571) fail to reject the null hypothesis of instrument validity, confirming that the instruments are not overidentified and are appropriately specified. Overall, the Lewbel IV-2SLS results validate the baseline findings. This substantially strengthens the causal interpretation of the main results.

Table 9 presents the marginal effects of CO₂ and N₂O emissions on LIFE at different percentiles of REC using the Lewbel IV-2SLS estimates. At low levels of REC (25th percentile), the adverse effects of CO₂ (−0.165) and N₂O (−0.156) emissions on LIFE are relatively strong. This suggests that countries with limited renewable energy integration experience more severe health deterioration from AIRP. At the median level of REC (50th percentile), the negative effects weaken to −0.103 for CO₂ and −0.096 for N₂O, indicating that increased renewable energy use begins to offset pollution-related health damages. At high renewable energy intensity (75th percentile), the harmful effects decline further to −0.054 for CO₂ and −0.048 for N₂O, though they remain statistically significant. This progressive reduction in magnitude clearly demonstrates the buffering role of renewable energy in protecting health from environmental degradation.

**Table 9:** Marginal effects estimates.

Percentile levels	Lewbel IV-2SLS	
	CO_2_	N_2_O
REC at P25	−0.165**	−0.156**
	(0.071)	(0.065)
REC at P50	−0.103**	−0.096**
	(0.045)	(0.041)
REC at P75	−0.054**	−0.048***
	(0.027)	(0.018)

P25, P50, and P75 are the 25th, 50th and 75th percentiles, respectively. ***p-values <0.01 and **p-value <0.05.

These IV-based marginal effects mirror the pattern obtained from the system GMM estimator (Table 7), confirming that the moderating role of renewable energy is robust to alternative identification strategies. The consistency across FE, system GMM, and Lewbel IV estimations strongly supports the conclusion that transitioning toward cleaner energy systems significantly attenuates the health costs of pollution in developing Asian economies.

**Figure 8:**
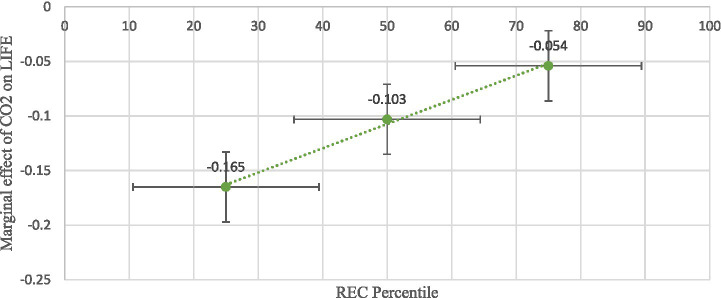
Marginal effects of CO_2_ emissions on life expectancy at different levels of renewable energy.

**Figure 9:**
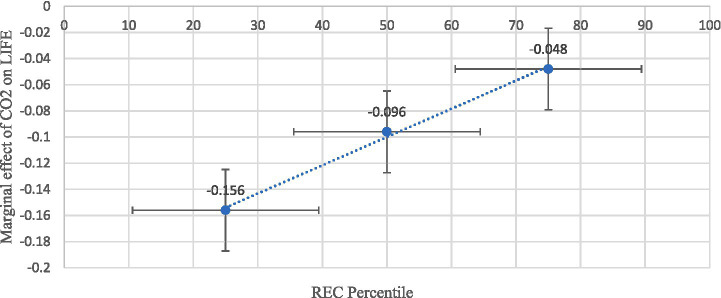
Marginal effects of N_2_O emissions on life expectancy at different levels of renewable energy.

Based on the Lewbel IV-2SLS estimation, Figures 8, 9 show that the negative effects of CO₂ and N₂O emissions on LIFE weaken as REC increases. At low REC levels (25th percentile), both emissions strongly reduce LIFE, but their adverse impacts diminish at median and upper-quartile levels. This confirms that higher adoption of renewable energy mitigates the health risks of emissions.

## 6 Discussions

This study provides compelling empirical evidence that AIRP exerts a significant negative effect on LIFE in developing Asian economies. Using FE, system GMM and Lewbel IV-2SLS estimators, the analysis confirms that higher levels of CO₂ and N₂O emissions substantially reduce LIFE, validating the first hypothesis (H1). Specifically, the results show that a unit increase in CO₂ and N₂O emissions reduces LIFE by approximately 0.311 and 0.198 years, respectively, underscoring that CO₂ exerts a more pronounced adverse impact. These findings are consistent with prior studies, such as Adebayo et al. (29), Uddin et al. (30), Kanat et al. (31), Azam and Adeleye (12), and Guo et al. (36), which similarly report a negative pollution–health relationship. The results reinforce the notion that in developing Asia, where rapid industrialization is accompanied by high fossil fuel dependency, the gains from economic growth are offset by environmental and health costs. Interestingly, the results diverge from studies like Mahalik et al. (35), Osei-Kusi et al. (34), and Wiredu et al. (33), who found a positive or neutral association between pollution and LIFE. These inconsistencies may stem from differences in data coverage, income levels, and pollution outsourcing effects, where wealthier economies externalize pollution-intensive production to developing regions. In contrast, most Asian developing economies lack such structural advantages, leading to a direct trade-off between industrial growth and health outcomes. The significant lag effect of LIFE further indicates strong persistence and path dependency in health improvements, implying that the consequences of current environmental policies will endure for decades.

The second hypothesis (H2) is also supported: renewable energy adoption significantly moderates the harmful impact of AIRP on LIFE. The negative and significant interaction terms (CO₂ × REC and N₂O × REC) suggest that the harmful effects of emissions diminish as renewable energy adoption rises. These findings align with Alharthi et al. (18), Aziz et al. (19), and Aziz et al. (42), who found that renewable energy mitigates pollution-induced damages and enhances environmental and health outcomes. The moderating mechanism operates through reduced reliance on non-renewable energy, technological innovation in clean energy systems, and the establishment of environmental regulations that collectively enhance air quality. However, the findings contrast with Li et al. (44) and Yi et al. (43), who documented that renewable energy initially correlates positively with pollution due to its limited scale and technological inefficiency in early development stages. This suggests that the health benefits of renewable energy adoption become more substantial only after a critical threshold of renewable penetration is achieved, an important insight for policy sequencing in developing countries. The marginal effects analysis further strengthens this conclusion by revealing heterogeneity across different levels of REC. At the 25th percentile of renewable energy use, AIRP’s negative effect on LIFE is pronounced; however, this effect declines substantially at the 75th percentile. For instance, the health damage from CO₂ decreases from 0.261 to 0.039 years as renewable energy intensity rises, while the N₂O-related loss declines from 0.165 to 0.054 years. This pattern highlights that scaling up renewable energy adoption can dramatically reduce the per-unit health costs of emissions, confirming renewable energy’s dual role as both an environmental and public health intervention.

The control variables further contextualize these relationships. Increased health expenditures and hospital bed capacity are associated with better health outcomes, indicating that investment in health infrastructure can partially offset environmental harms. Conversely, fossil fuel energy consumption and population density exert negative effects, reflecting the combined pressures of pollution exposure and overburdened healthcare systems. GDP per capita shows a significant relationship, suggesting that economic growth alone, without structural shifts toward cleaner energy, cannot guarantee improved life span. Overall, this study contributes novel evidence by integrating renewable energy as a moderating factor in the AIRP–LIFE nexus, a dimension largely overlooked in prior research. It not only strengthens the empirical basis for renewable energy as a climate mitigation tool but also extends its relevance to the public health domain. These findings imply that expanding renewable energy portfolios is not merely an environmental necessity but a public health imperative.

The findings of this study should be interpreted in light of several limitations. First, AIRP is proxied using CO₂ and N₂O emissions, which primarily reflect pollution-intensive production and energy systems rather than direct ambient exposure to harmful pollutants such as PM₂.₅ or ozone. While these indicators are widely used in cross-country analyses due to data availability and comparability, they cannot capture spatial variation in exposure or short-term pollution spikes that are central to epidemiological assessments. Second, the absence of consistent PM₂.₅ or ozone data across developing Asian economies constrains the ability to draw precise exposure response inferences. As such, the estimated effects should be interpreted as reflecting long-run health consequences of pollution-intensive economic structures rather than direct causal effects of ambient pollutant concentrations. Despite these limitations, the study contributes valuable macro-level evidence on the health implications of pollution and energy transitions in developing Asia.

Although regional heterogeneity is not explicitly examined in this study, the findings provide an average effect across developing Asian economies, offering a useful benchmark for future region-specific investigations. The findings underscore the need for developing Asian economies to accelerate their renewable energy transition to achieve long-term health and sustainability goals. Future research should disaggregate renewable energy sources such as solar, wind, hydro, and biomass to capture their heterogeneous environmental and health impacts. Moreover, considering potential endogeneity between renewable energy policies and institutional quality could yield deeper causal insights. At the policy level, integrating health and energy planning, strengthening environmental governance, and targeting urban pollution hotspots through clean energy investments can jointly enhance human welfare and environmental quality. As such, renewable energy should be viewed as a multidimensional policy instrument, advancing environmental sustainability, improving public health, and supporting progress toward SDG 3 (health), SDG 7 (clean energy), and SDG 13 (climate action).

## 7 Conclusion and policy implications

### 7.1 Conclusion

This study examines the impact of air pollution on life expectancy in 30 developing Asian economies over the period 2000–2023, emphasizing the moderating role of renewable energy adoption. Using fixed effects, system GMM, and Lewbel IV-2SLS estimators, the findings reveal robust and consistent evidence that higher levels of CO₂ and N₂O emissions significantly reduce life expectancy. Among these pollutants, CO₂ exerts a stronger and more persistent detrimental effect, highlighting that fossil fuel dependency remains a key barrier to sustainable human development in rapidly industrializing economies. By incorporating renewable energy as a moderating variable, this study advances the conventional air pollution–health nexus and provides novel evidence that clean energy transition can effectively mitigate the adverse health impacts of pollution. The findings indicate that as the share of renewable energy in total energy consumption increases, the negative effect of emissions on life expectancy weakens considerably. This confirms that renewable energy not only contributes to environmental sustainability but also serves as a health instrument, reducing the disease burden associated with pollution exposure and improving life span. The marginal effects analysis further demonstrates that the health cost of each additional unit of CO₂ or N₂O emission declines as the share of renewable energy increases, highlighting renewable energy’s pivotal role in improving population well-being.

The persistence of life expectancy over time further indicates that the health outcomes of current environmental and energy policies are cumulative, underscoring the long-term consequences of today’s policy choices. Control variables, including health expenditures, hospital capacity, and income levels, play an enabling role in enhancing resilience against pollution-related health risks, whereas reliance on fossil fuels and rapid population growth aggravate them. Overall, the study concludes that renewable energy expansion provides a dual dividend for developing Asia: it promotes ecological balance while safeguarding human life. These findings have profound implications for achieving the SDGs, particularly SDG 3 (Good Health and Well-being) by improving life expectancy, SDG 7 (Affordable and Clean Energy) through clean energy transition, and SDG 13 (Climate Action) by curbing greenhouse gas emissions. Integrating renewable energy policies with public health strategies thus represents a sustainable pathway toward resilient, low-carbon, and health-secure societies in developing Asia.

### 7.2 Policy implications

The empirical findings of this study underscore that improving air quality and expanding renewable energy are central to enhancing life span and achieving sustainable development in developing Asian economies. Based on the results, several key policy implications emerge. First, governments must integrate air quality management into national public health and development strategies. The evidence that CO₂ and N₂O emissions significantly reduce life expectancy highlights the urgency of adopting stricter emission control standards, phasing out heavily polluting fuels, and enforcing compliance with environmental regulations. Recognizing clean air as a determinant of population health directly contributes to SDG 3 (Good Health and Well-being) by reducing the disease burden linked to pollution-related mortality and morbidity.

Second, expanding renewable energy capacity should be prioritized as both an environmental and health policy tool. The moderating role of renewable energy in mitigating pollution-induced health deterioration implies that transitioning toward solar, wind, and other clean technologies can yield simultaneous environmental and health dividends. To achieve SDG 7 (Affordable and Clean Energy), policymakers should promote fiscal and regulatory incentives such as feed-in tariffs, tax credits, and low-interest green financing to attract private investment in renewable energy infrastructure. Moreover, expanding decentralized renewable energy systems in rural and underserved regions can improve both energy equity and public health outcomes. Third, reducing fossil fuel dependence is crucial for achieving climate and health co-benefits. Phasing out coal and petroleum subsidies and redirecting these resources toward clean energy innovation and public health infrastructure will accelerate the green transition while protecting vulnerable populations. Such measures are directly aligned with SDG 13 (Climate Action), as they lower greenhouse gas emissions and enhance climate resilience.

### 7.3 Limitations and directions for future research

This study provides robust evidence on the impact of air pollution on life expectancy in developing Asian economies, several avenues for future research remain. First, the analysis is conducted at an aggregate regional level and does not explicitly explore heterogeneity across Asian sub-regions such as South Asia, East Asia, and Central Asia. Given substantial differences in economic structures, energy mixes, institutional capacity, and pollution exposure across these regions, future studies could undertake sub-regional or country-group analyses to uncover region-specific dynamics and support more targeted public health and environmental policies.

Second, while the effects of industrialization are captured indirectly through emission indicators, energy consumption, and income growth, future work could explicitly model the industrialization–pollution–health nexus. Incorporating direct measures of industrialization, such as industrial value added, manufacturing intensity, or urban industrial concentration, would allow for a more detailed examination of transmission mechanisms linking economic transformation, environmental quality, and public health.

Finally, the AIRP is measured using CO₂ and N₂O emissions due to their wide availability and cross-country comparability. While primarily greenhouse gases, they serve as effective proxies for fossil fuel–intensive energy systems and production patterns that also produce health-relevant co-pollutants like PM₂.₅, nitrogen oxides, and sulfur dioxide. Thus, these indicators capture long-term exposure to pollution-intensive economic activity, offering valuable insights for public health. Although direct PM₂.₅ or ozone data are limited across countries, future studies could enhance assessment by integrating satellite-based or ground-level air quality measurements where available.

## Data Availability

Publicly available datasets were analyzed in this study. This data can be found here: World Development Indicators (https://databank.worldbank.org/source/world-development-indicators).
